# Effects of drugs on the oxygen dissociation curve—a scoping review

**DOI:** 10.1007/s00228-024-03781-8

**Published:** 2024-11-25

**Authors:** Thomas Haller, Lukas Lesser, Simon Woyke

**Affiliations:** 1 Department of Physiology and Medical Physics, Institute of Physiology, Medical University of Innsbruck, Innsbruck, Austria; 2 Department of Anaesthesiology and Intensive Care Medicine, Medical University of Innsbruck, Innsbruck, Austria

**Keywords:** Pharmaceuticals, Hemoglobin, Oxygen affinity, Half saturation pressure, P_50_

## Abstract

**Purpose:**

The shape and position of the hemoglobin-oxygen dissociation curve (ODC) is of critical importance in medicine, as it determines the uptake of O_2_ in the lungs and the delivery of O_2_ to the tissues. Numerous reports have identified affinity-modulating effects of drugs in humans. Such effects may be relevant to conditions such as pulmonary diffusion disorders, peripheral vascular disease, or coronary artery disease. The aim of this scoping review was to assess and summarize the current evidence on these effects.

**Methods:**

The review was based on the PRISMA-ScR guidelines. We searched PubMed and the Cochrane Library and only included papers with free full-text access. The search covers all papers published before September 2024 and used the following keywords: “Oxygen affinity” or “oxygen dissociation curve” in combination with > 100 substance classes that should cover most drugs in clinical use.

**Results:**

The search returned 2447 hits of which 80 were selected for further full text review. In terms of discipline, cardiology accounted for the largest proportion, and in terms of effect quality, a right-ward shift resulting in improved tissue oxygenation was most common. For example, quantitative data show an increase in P_50_ of 6.1–12.4% and 25–53% for propranolol and RSR13, respectively.

**Conclusion:**

Despite a substantial body of data, the effects of the vast majority of drugs on the ODC have not been studied or have not been studied in sufficient detail. The undeniable potential for medical interventions to alter the ODC calls for revival of this area of research.

**Supplementary Information:**

The online version contains supplementary material available at 10.1007/s00228-024-03781-8.

## Introduction

Oxygen transport to our tissues consists of two convective and two diffusive pathways connected in series: alveolar ventilation (convection), O_2_ diffusion across the alveolar-capillary membrane, blood flow to the microcirculation (convection), and finally diffusion of O_2_ into the cell’s furnaces, the mitochondria. Thus, total oxygen supply to cells is critically dependent on all 4 processes, with the reversible binding of oxygen to hemoglobin (Hb) receiving probably the most attention in the history of blood and respiratory physiology. This reversible binding was first observed by Stokes in 1864 [1], long before the sigmoidal shape of the oxygen dissociation was reported by Bohr et al. in 1904 [2]. Nearly a decade later, an idea of the puzzling reason for this non-linear shape, namely, some kind of cooperative behavior in O_2_ binding between Hb subunits, first came up with the work of A.V. Hill in 1913 [3] and was then confirmed by numerous experimental work that followed in the course of the century. Some allosteric modulators of the oxygen dissociation curve (ODC) were already known at that time: CO_2_ (by Bohr, Hasselbalch, and Krogh in 1904 [2]), H^+^ (by Barcroft and Orbeli in 1910 [4]), and temperature (Barcroft and King in 1909 [5]). A fourth and most potent, 2,3-bisphosphoglycerate (2,3-BPG), was discovered almost half a century later and almost simultaneously by Benesch and Benesch in 1967 [6] and Chanutin and Curnish in the same year [7]. 2,3-BPG, produced in the Rapoport-Lübering bypass of glycolysis in red blood cells, is a major regulator of oxygen release by Hb. All of these four parameters shift the ODC to the right, meaning that Hb loses its affinity to interact with O_2_ as it passes through a metabolically active tissue.

The shape and position of the ODC are undoubtedly of utmost importance in physiology [8]. In the steep part of the curve, there is efficient unloading of O_2_ from Hb with respect to small changes in tissue PO_2_, whereas in the flat, asymptotic part, which is characteristic of the gas situation in the lung, O_2_ binding is almost independent of PO_2_. Any change in the position of the ODC will therefore profoundly alter O_2_ supply to the cells and O_2_ uptake in the lungs, at the same time, although differently: A left-shifted curve facilitates O_2_ binding whenever alveolar PO_2_ is reduced but impairs O_2_ delivery to the cells. A right-shifted curve improves tissue oxygenation but may be limiting O_2_ uptake in the lungs. A quantitative measure of the position of the ODC is the P_50_, the partial pressure of O_2_ at 50% O_2_ saturation of Hb. A measure of the steepness of the ODC around its P_50_ is the Hill coefficient *n*, defined as log (SO_2_/1-SO_2_)/log PO_2_. The higher this ratio, the higher the degree of cooperativity between the four subunits of the tetrameric Hb molecule in terms of O_2_ binding. In other words, the higher *n*, the more sensitive is Hb oxygenation/deoxygenation to PO_2_ at low oxygen regimes.

The issue of left- versus right-shifted ODCs has been debated for long, and the benefits of either position in special situations, such as high-altitude stays, are still unresolved [9]. Moreover, given the enormous impact of modulating oxygen delivery to a critically ill patient suffering from respiratory failure (supply) or tissue hypoxia (demand), it is remarkable that this issue has barely found its way into medical research or practice. It was during the COVID pandemic that Böning et al. published a paper that critically addressed the urge to identify affinity modulating drugs to cope with an aggravated O_2_ situation, especially in SARS-CoV-2 infected patients [10, 11]. In addition, in all situations of oxygen delivery deficiency, such as hypoxemia, pulmonary diffusion disorders, peripheral and central arterial diseases, coronary artery disease, and various forms of tissue ischemia, the ODC and its intended or accidental manipulation by administered drugs may play an underestimated role in clinical practice.

The aim of this scoping review was therefore to evaluate and summarize the current knowledge on the effects of various drugs on the ODC. This topic has not yet been systematically summarized but consists of a number of highly fragmented and mostly outdated reports and casual references. The review was designed as a scoping review based on the PRISMA-ScR guidelines, as introduced by Tricco et al. [12].

## Methods

### Data sources

PubMed and the Cochrane Library were used for the search process. To be considered for this review, papers had to meet the following criteria: They are available in at least one of two online directories, the language is English or German, and a full-text version is available through our institutional subscriptions (see limitations). Studies that did not provide sufficient information or consisted only of an abstract were excluded. If a study was considered unusable due to other reasons, it was noted in our documents. The first search was performed in July 2023 and the last one in September 2024. There were no restrictions regarding the publication date of a study.

### Study selection

**Fig. 1:**
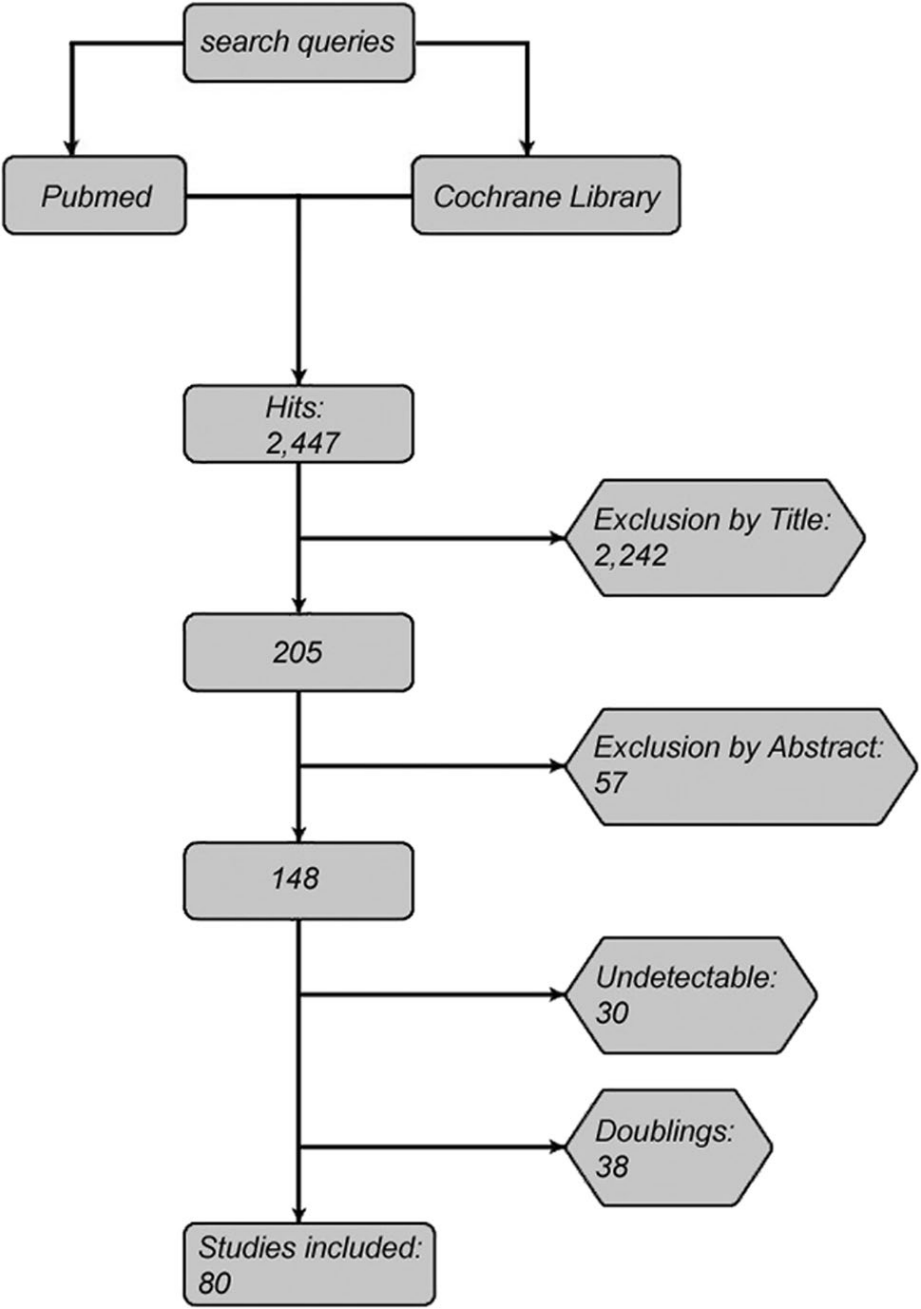
Flowchart of the stereotypical search query

First, the search terms were defined. The drugs included in the search were obtained from the German website “Gelbe Liste Pharmindex,” a database for medicine and pharmacology. The search terms for all drugs were then grouped into drug classes, such as “ACE-Inhibitors.” Drug classes were used because a search for the trade names of pharmaceuticals would probably be unmanageable. The drug classes screened are listed in Table 1. All search processes were performed in the same, stereotypical way, namely, [1] and [2], where [1] was either “oxygen affinity” in title and/or abstract or “oxygen dissociation curve” in title and/or abstract. These two terms seemed to be the most appropriate to find studies on drug effects on the ODC. We validated the search strategy by comparing the retrievals with a handful of papers on ODC modulators that we had already collected in the past. Number [2] were the substance classes mentioned above. All different combinations were used in both databases, PubMed and Cochrane Library. The search performed in this way resulted in 2447 hits, which were entered into an Excel spreadsheet containing, among other entries, the date of the search, the database that provided the result, the combination of search terms used, and the number of results which were provided by the database. The actual sighting started with an exclusion by title followed by an exclusion by abstract. The former significantly reduced the number of potentially eligible papers to 205. Of the remaining studies, 57 were excluded by abstract. Doubtful cases were discussed among all authors. Of the remaining 148 studies, 30 studies had to be excluded because they were out of reach. In addition, 38 studies were in duplicate. After this process, 80 studies remained for full-text review. The flowchart in Fig. 1 illustrates the whole process. Finally, studies were then grouped according to the field of specialization in which the investigated drug is mainly used (Tables 2, 3, 4, and 5).

**Table 1:**
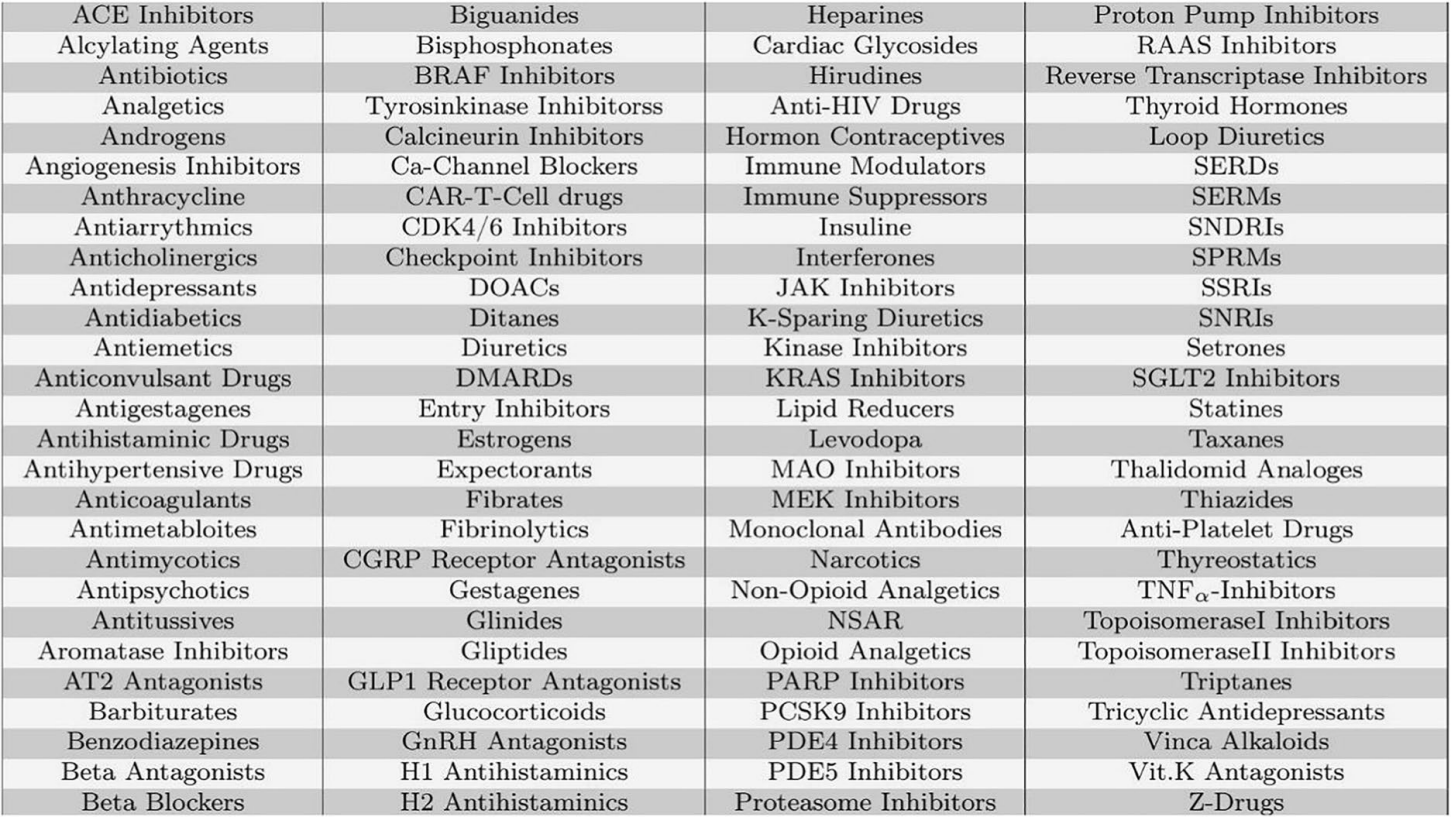
Substance classes used for the systematic search. Substance classes were taken from the website “Gelbe Liste Pharmindex”

## Results

### Characteristics of sources of evidence

**Fig. 2:**
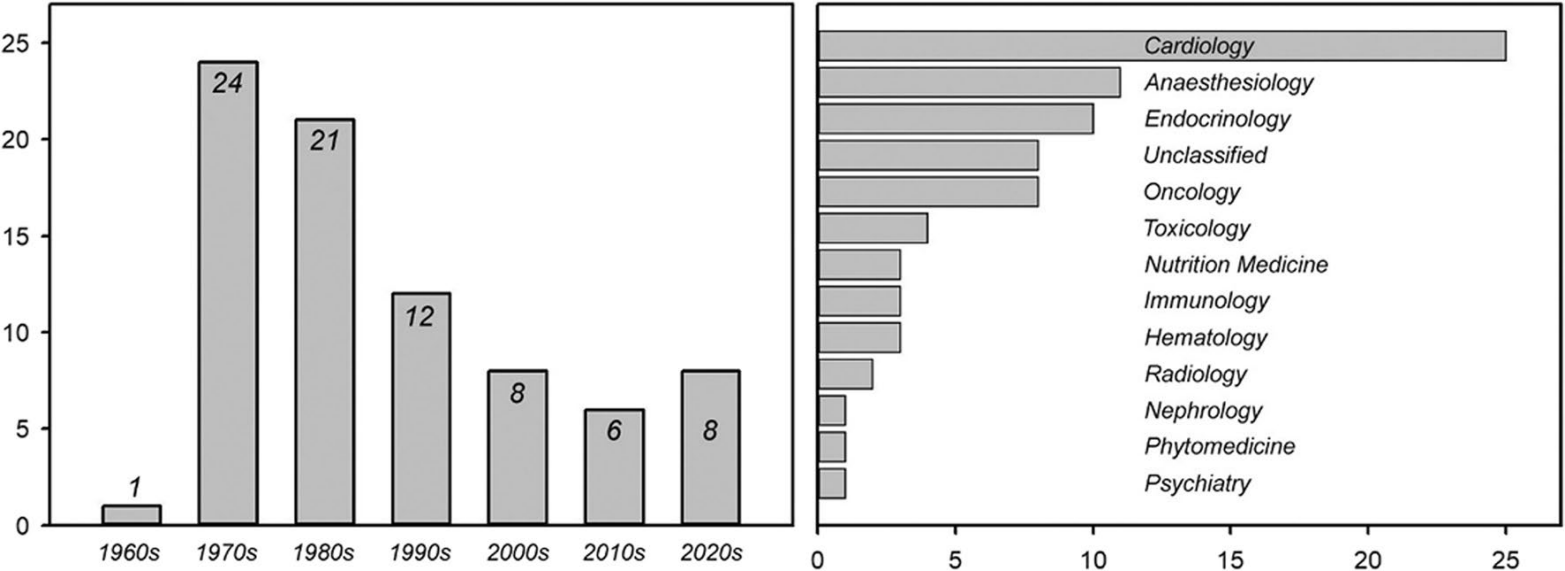
Number of studies by decade (left) and by discipline (right)

**Fig. 3:**
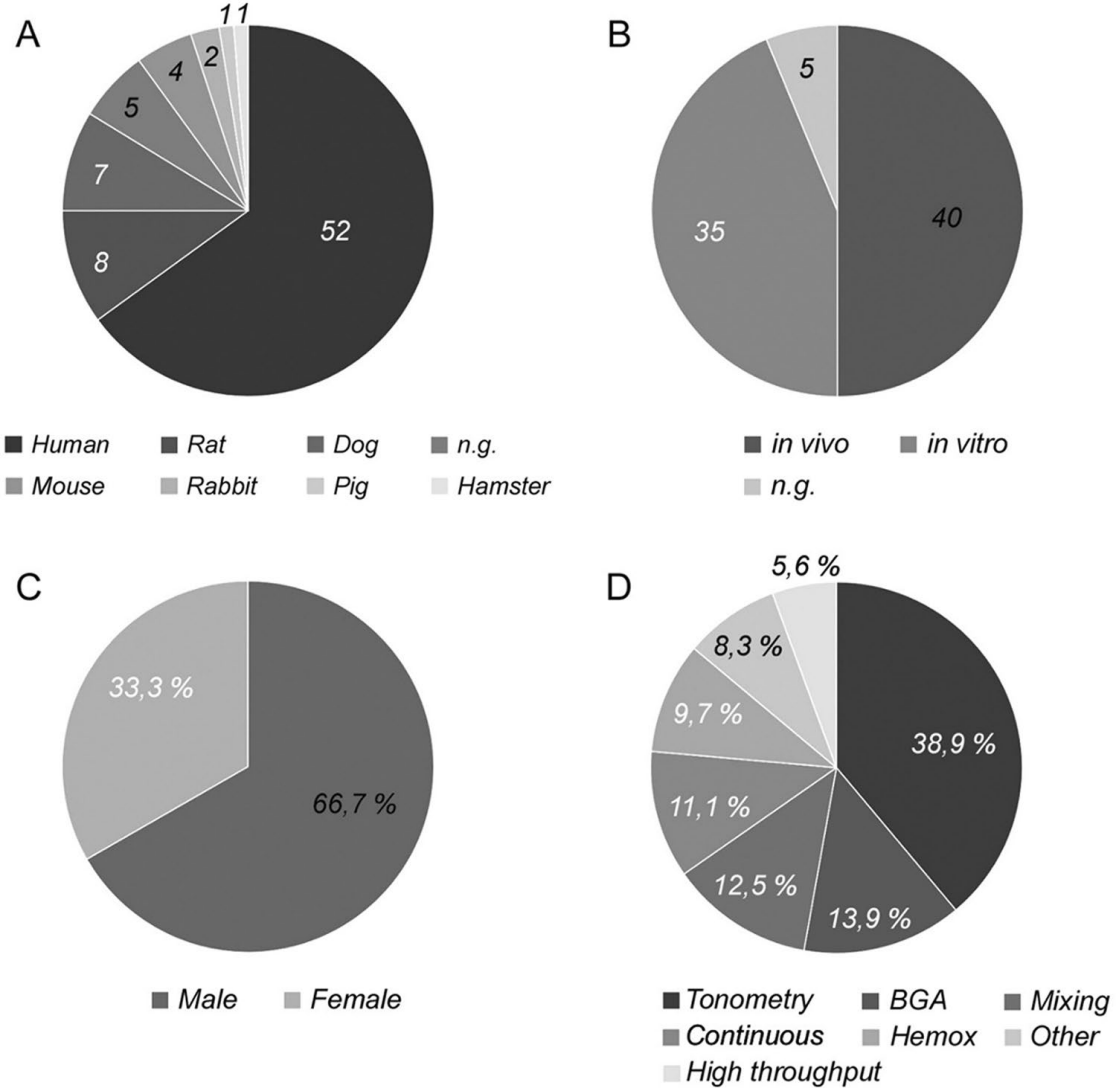
Number of studies by test organisms (**A**), by test settings (**B**), and by the methods used to measure or calculate the ODC (**D**). **C** Percent distribution of studies between females and males

Time range: The distribution of studies by date of publication is shown in Fig. 2. Interestingly, the majority of them were published between 1970 and 1989. In later years, the numbers decreased. Our search query returned no results for the period before 1960 (see “Discussion”). Disciplines: The included studies were grouped according to the disciplines in which the drugs are mainly used (Fig. 2). Cardiology is clearly ahead of all other disciplines, followed by anaesthesiology and endocrinology. Some of the drugs are not associated with a certain discipline (“unclassified”). That is mostly the case when they are still in the early stages of testing, not yet on the market, or no longer in use. Test organisms: The vast majority of studies were conducted on humans, followed by rats and dogs (Fig. 3). A notable proportion of five studies did not specify the test organism. Test setting: In vivo studies dominate but are roughly balanced with in vitro approaches (Fig. 3). A surprising number of five studies did not specify their approach at all. Sex distribution: Of the 80 studies reviewed, only 24 provided information about the sex of the participants. Of these, exactly two-thirds of studies were conducted on men (Fig. 3). Methods: Fig. 3 also gives an overview of the methods used. The most popular is a tonometric approach, followed by blood gas analyzers (BGA), the mixing technique, continuous methods, and the Hemox Analyzer, all of which are briefly described in the “Discussion”.

The results of the individual sources of evidence, grouped by area of specialization, are listed in Tables 2, 3, 4, and 5. All abbreviations used in these tables and further explanations of the entries can be found in the legend of Tables 2, 3, 4, and 5.

**Table 2:**
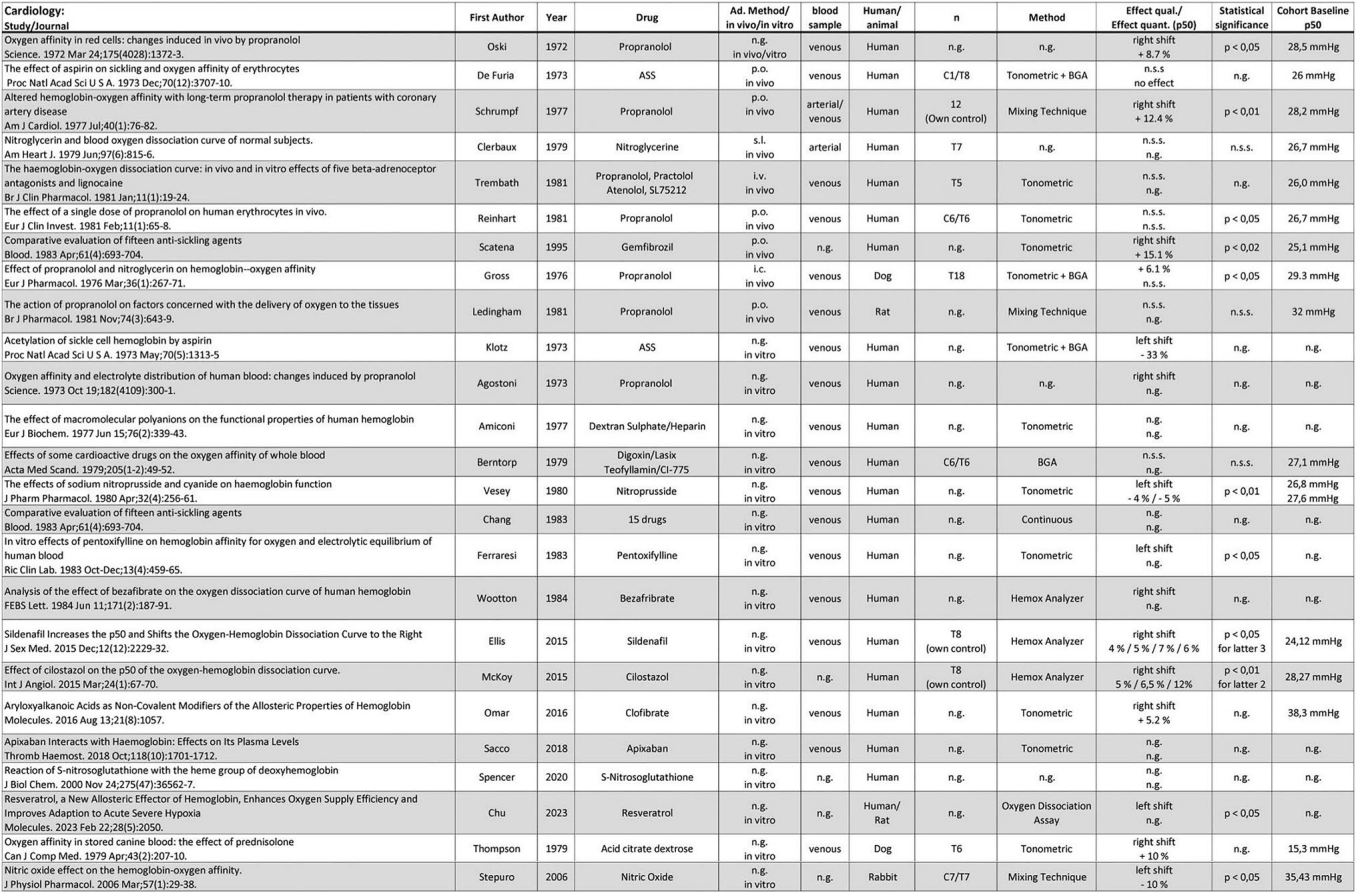
Results of individual sources of evidence, grouped into 12 specific disciplines (plus 1 “unclassified”). The order of publications within the disciplines is in vivo/human, in vivo/animal, in vitro human, and in vitro animal. Within these groups, the order is by year of publication (ascending). Abbreviations used: *Ad.*, administration; *C*, control group; *i.a.*, intraarterial; *i.c.*, intracutaneous; *i.m.*, intramuscular; *i.p.*, intraperitoneal; *i.v.*, intravenous; *n*, number of experiments; *n.g.*, not given; *n.s.s.*, not statistically significant; *p.o.*, per os; *qual.*, quality; *quant.*, quantity; *s.c.*, subcutaneous; *s.l.*, sublingual; *T*, test group. The percentage in column “Effect quant. (P_50_)” indicates the percentage change in the P_50_. Numbers next to C or T refer to the number of individuals in the control and test group, respectively. “Own control” denotes the same individuals for test and control. *Opinion papers or reviews

**Table 3:**
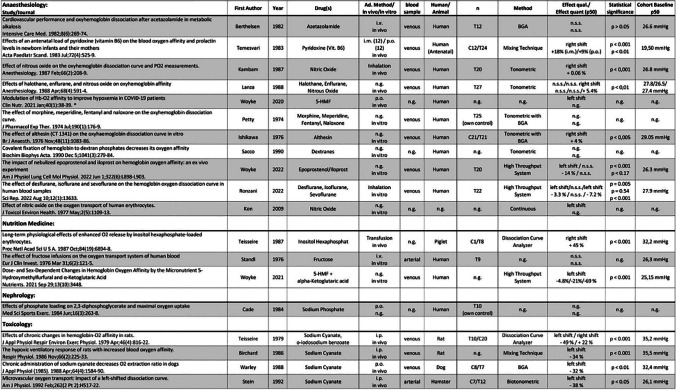
Results of individual sources of evidence, grouped into 12 specific disciplines (plus 1 “unclassified”). The order of publications within the disciplines is in vivo/human, in vivo/animal, in vitro human, and in vitro animal. Within these groups, the order is by year of publication (ascending). Abbreviations used: *Ad.*, administration; *C*, control group; *i.a.*, intraarterial; *i.c.*, intracutaneous; *i.m.*, intramuscular; *i.p.*, intraperitoneal; *i.v.*, intravenous; *n*, number of experiments; *n.g.*, not given; *n.s.s.*, not statistically significant; *p.o.*, per os; *qual.*, quality; *quant.*, quantity; *s.c.*, subcutaneous; *s.l.*, sublingual; *T*, test group. The percentage in column “Effect quant. (P_50_)” indicates the percentage change in the P_50_. Numbers next to C or T refer to the number of individuals in the control and test group, respectively. “Own control” denotes the same individuals for test and control. *Opinion papers or reviews

**Table 4:**
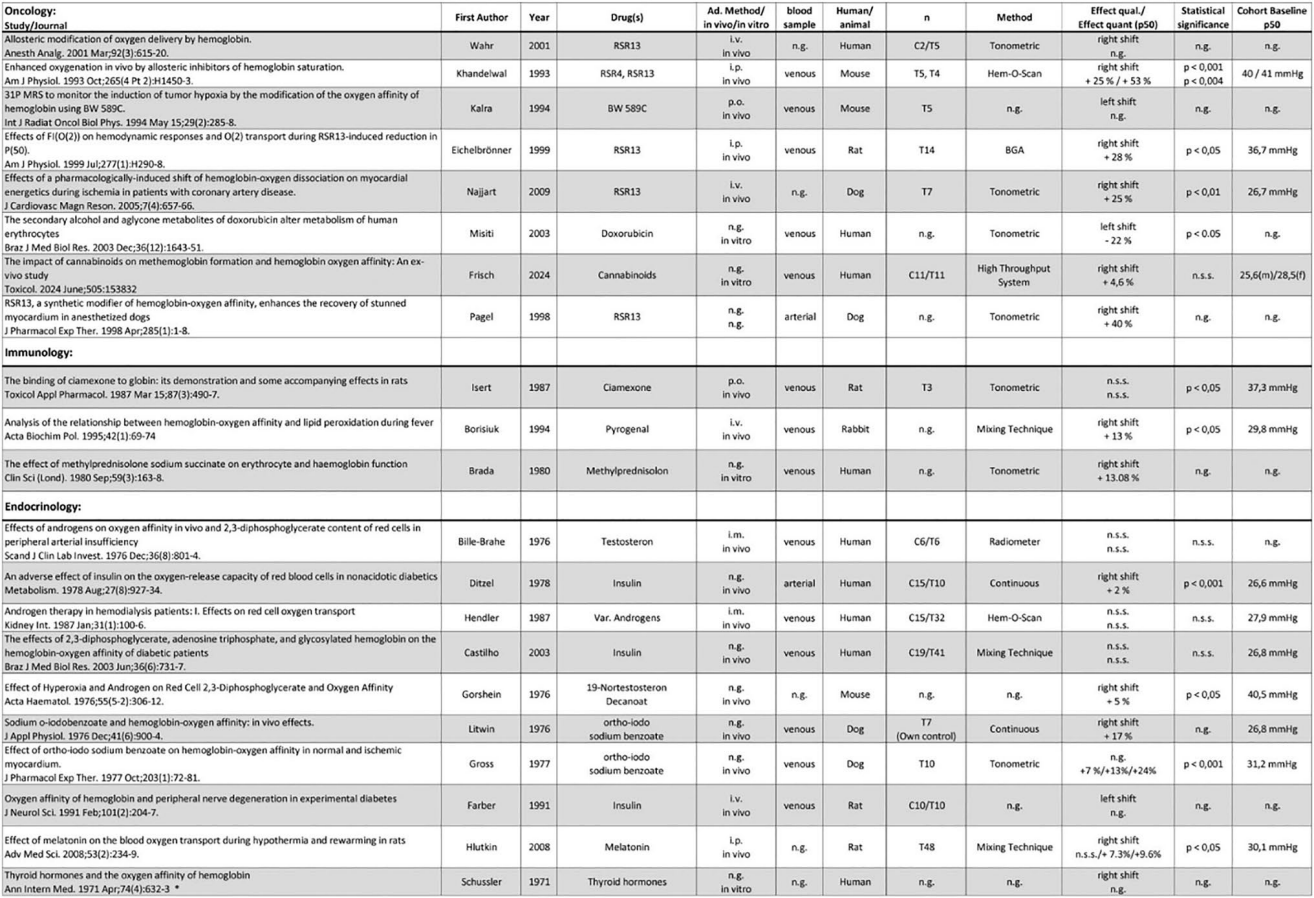
Results of individual sources of evidence, grouped into 12 specific disciplines (plus 1 “unclassified”). The order of publications within the disciplines is in vivo/human, in vivo/animal, in vitro human, and in vitro animal. Within these groups, the order is by year of publication (ascending). Abbreviations used: *Ad.*, administration; *C*, control group; *i.a.*, intraarterial; *i.c.*, intracutaneous; *i.m.*, intramuscular; *i.p.*, intraperitoneal; *i.v.*, intravenous; *n*, number of experiments; *n.g.*, not given; *n.s.s.*, not statistically significant; *p.o.*, per os; *qual.*, quality; *quant.*, quantity; *s.c.*, subcutaneous; *s.l.*, sublingual; *T*, test group. The percentage in column “Effect quant. (P_50_)” indicates the percentage change in the P_50_. Numbers next to C or T refer to the number of individuals in the control and test group, respectively. “Own control” denotes the same individuals for test and control. *Opinion papers or reviews

**Table 5:**
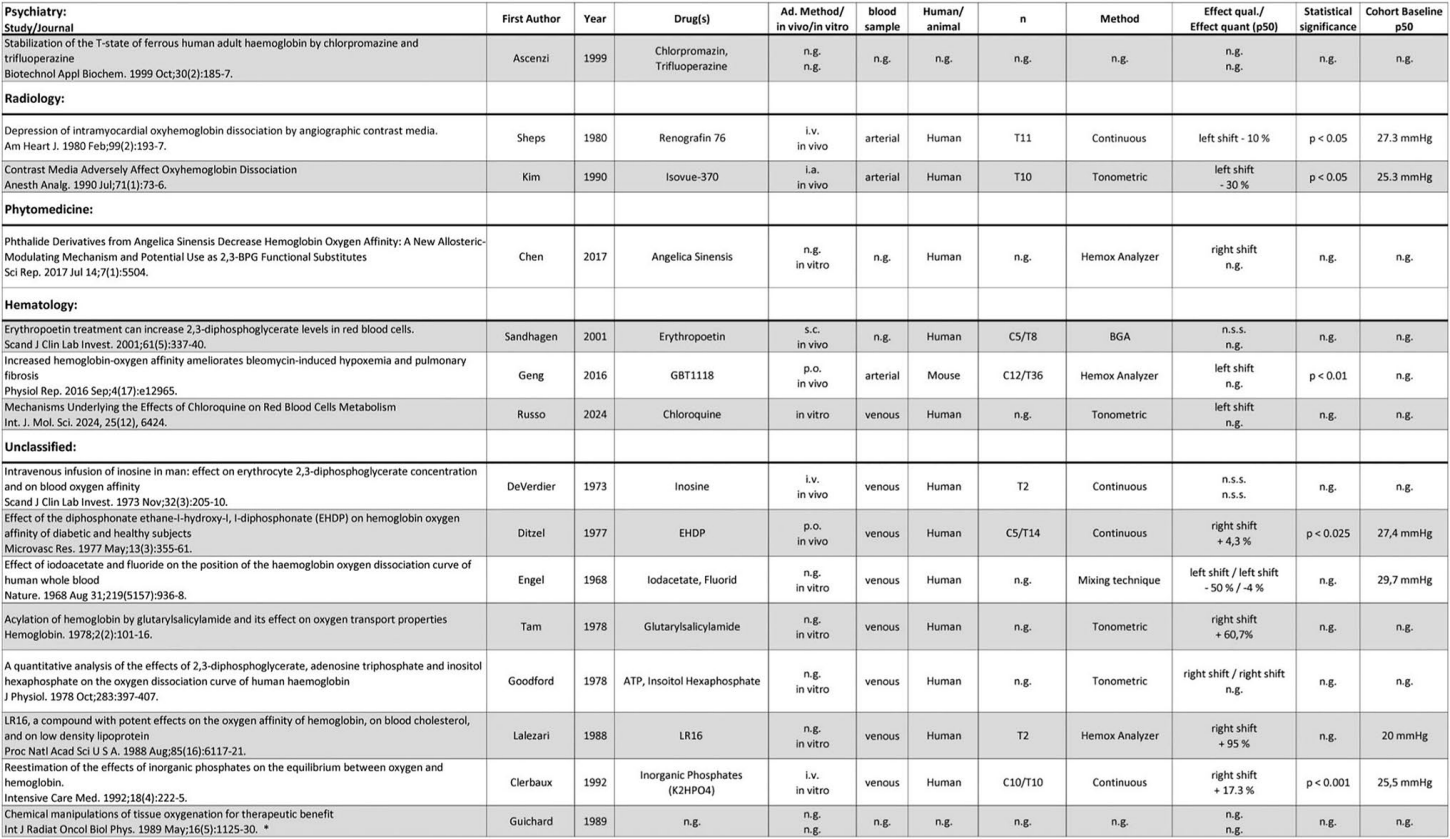
Results of individual sources of evidence, grouped into 12 specific disciplines (plus 1 “unclassified”). The order of publications within the disciplines is in vivo/human, in vivo/animal, in vitro human, and in vitro animal. Within these groups, the order is by year of publication (ascending). Abbreviations used: *Ad.*, administration; *C*, control group; *i.a.*, intraarterial; *i.c.*, intracutaneous; *i.m.*, intramuscular; *i.p.*, intraperitoneal; *i.v.*, intravenous; *n*, number of experiments; *n.g.*, not given; *n.s.s.*, not statistically significant; *p.o.*, per os; *qual.*, quality; *quant.*, quantity; *s.c.*, subcutaneous; *s.l.*, sublingual; *T*, test group. The percentage in column “Effect quant. (P_50_)” indicates the percentage change in the P_50_. Numbers next to C or T refer to the number of individuals in the control and test group, respectively. “Own control” denotes the same individuals for test and control. *Opinion papers or reviews

## Discussion

Our search yielded less than a hundred studies on this topic. This is comparatively small considering the number of drugs in clinical use worldwide or in current development for clinical use. Thus, the vast majority of drugs routinely administered to humans have not been studied for their effects on Hb oxygen transport. In addition, we found that a significant proportion of the reviewed publications were of poor study quality, with many providing little or no information on statistics, number of subjects, methods, or even test organisms. Other studies suffer from small numbers of participants or test runs that are unlikely to produce statistically significant results. Unfortunately, many studies did not report the distribution of male and female participants, and when they did, males were overrepresented in most cases. This is a notable omission given that the P_50_ values are significantly different between women and men [13]. In addition, there is a remarkable proportion of studies in which the authors declared a conflict of interest (e.g., paid by the manufacturer of the product), which may also limit comparability of the results. Finally, a large number of studies are outdated and deal with substances that in some countries are no longer used or recommended in clinical use and have been replaced by others. This is the case, for example, for propranolol, a ß-blocker and class II antiarrhythmic used in cardiology with a significant right-ward shift effect on the ODC shown in several studies. This effect is discussed to be due to the release of 2,3-BPG from the erythrocyte membrane and binding to Hb, thereby reducing the oxygen affinity of Hb [14]. This right-ward shift of the ODC might enhance oxygen extraction at the tissue level. However, propranolol has been replaced in many intensive care units for the treatment of arrhythmia by short acting ß-blockers like esmolol or landiolol, for which there are no data on potential effects on oxygen binding to Hb. This is another example of why further studies in this area are urgently needed.

There may be many reasons for this unsatisfying study situation. We speculate that the limited accessibility of appropriate analytical methods necessary to perform an ODC measurement is one of them. For example, the Hemox Analyzer is only available in a few institutions because it is quite expensive for a single-purpose device. Other methods or devices (except blood gas analysis) are not commercially available and would have to be custom-built, which could prevent many researchers from carrying out specific projects. In addition, the vast majority of methods suffer from the limitation that measurements in single cuvette systems, such as the Hemox Analyzer, are cumbersome, time-consuming, and do not provide the ability to run controls side by side. Another reason for the paucity of studies may be that the interest in the ODC has steadily declined since the 1970s and 1980s, as analyzed in Fig. 2. We can only speculate, but perhaps the impact of the ODC in a clinical setting has been overlooked, or people are not really aware of its significance.

**Fig. 4:**
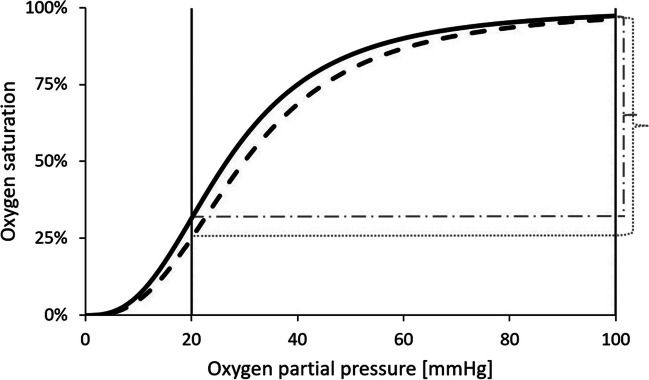
Standard ODC (black sigmoidal curve) and ODC with a 12.5% increase in P_50_ (dashed curve). Given a tissue PO_2_ of 20 mm Hg (left vertical line), O_2_ extraction (curly brackets) is greater in the right-shifted ODC. For details, see Text

The importance of the ODC has already been mentioned in the introduction but should be underscored by the following examples: The working myocardium has an exceptionally high oxygen extraction rate and is very vulnerable in ischemic and hypoxic situations. Right- or left-ward shifted ODCs, resulting in either increased (right-ward shifted) or decreased (left-ward shifted) tissue oxygenation, would have a significant impact on heart functionality, especially in any kind of diseased state when oxygen delivery is generally low. For example, Lucas et al. injected 5-hydroxymethylfurfural (5-HMF) or only vehicle to hamsters [15]. The 5-HMF induced left-ward shift of the ODC resulted in improved cardiac indices, stroke volume, cardiac output, ejection fraction, and stroke work compared to control group when exposed to hypoxia. On the other hand, Watanabe and colleagues showed that mice with severe heart failure and treated with the oxygen affinity modulator RSR13 (which induces a right-ward shift) had improved treadmill running performance due to increased oxygen delivery in skeletal muscle [16]. P_50_ was increased by 12.5% in their experiments. In humans, a right-ward shift of the ODC by the same amount would increase P_50_ from a normal value of 26.7 (Fig. 4; black line) to 30.0 mmHg (dashed line). Assuming normoxia and normal pulmonary oxygenation, Hb is almost completely saturated in both cases (SO_2_ 97.3% vs. SO_2_ 96.3%; right vertical solid line), while at the tissue level, assuming a PO_2_ of 20 mmHg (left vertical solid line), SO_2_ is lower in the right-shifted ODC and correspondingly more O_2_ is extracted (SO_2_ 31.4% vs. SO_2_ 25%). In this example, the oxygen extraction rate (O_2_ER = (SaO_2_—SvO_2_) / SaO_2_); a = arterial, v = venous) is 74.0% compared to 67.7%. Further assuming a Hb concentration of 150 g/l and using a Hüfner number of 1.34 ml O_2_/g Hb, this would mean that for every liter of cardiac perfusion, an additional ~ 11 ml of pure O_2_ would be available to the cells. Although we are not aware of a human cohort study demonstrating a direct physiological effect on patients with this additional amount of O_2_, all cardiologists would probably agree that anything that improves tissue oxygenation and prevents tissue hypoxia should be beneficial to a critically ill patient at some point. In a case report by Al-Qudsi et al., two patients with persistent severe hypoxic respiratory failure were treated with the anti-sickling agent voxelotor [17]. This drug stabilizes Hb in its oxygenated state by inducing a left-ward shift of the ODC. During the treatment, the oxygen saturation to FiO_2_ ratio increased substantially, reducing the invasiveness of mechanical ventilation. Unfortunately, the change in tissue oxygenation by this left-shifted ODC was not investigated in this study.

Our search revealed a handful of methods for recording an ODC, and the most common ones are briefly described here. First and foremost is a tonometric approach, where blood samples are successively exposed to ≥ 2 different gas mixtures with predefined PO_2_, while SO_2_ is determined by dual wavelength absorption measurements. Continuous methods, in which the oxygen content of the gas phase is constantly decreasing or increasing, require the measurement of PO_2_ and SO_2_ in the sample at certain time intervals. Continuous methods are largely based on the method described by Duvelleroy and colleagues, where a known volume of deoxygenated blood is exposed to a known volume of O_2_ and PO_2_ and oxygen content are measured [18]. In the mixing technique, blood samples are divided into two aliquots, one exposed to an oxygen-rich gas mixture (SO_2_ = 100%) and the other to an oxygen-free gas (= SO_2_ 0%). These two aliquots are then mixed in predefined volume fractions to obtain aliquots of known SO_2_. PO_2_ is then measured in these aliquots. In vivo studies are usually based on blood gas analysis, where PO_2_ and SO_2_ are measured from a blood sample and the P_50_ is then extrapolated based on a predefined algorithm [19]. Finally, the Hemox Analyzer is a commercially available instrument (TCS Scientific Corp., PA, USA) that has been on the market for more than 40 years. It determines the ODC by exposing 2–50 µl of whole blood or hemolysate, diluted in 5 ml of buffered, anti-foaming solution, to a decreasing partial pressure of oxygen in an optical cuvette under constant agitation. The change in oxygen tension is detected by a Clark oxygen electrode inside the cuvette, while the decrease in oxyhemoglobin fraction (% HbO_2_) is simultaneously monitored by dual wavelength spectroscopy at 560 nm and 576 nm, respectively. Recently, high-throughput assays have been developed that allow simultaneous measurements of large numbers of samples in microplate reader instruments [20, 21].

Our search has limitations that should be addressed here: First, our scoping review may not be fully replicable by all researchers, as it was limited to publications accessible through our institutional subscriptions. This limitation is important, as different institutions have varying agreements with publishers, and these agreements change over time (see Supplementary Information for the full list of journals accessible through our library). Second, our review does not claim to be exhaustive. Each search mask returns results based on its specific criteria, meaning that papers without the defined keywords may be missed. To avoid this, one would need to define more search forms, which, however, would significantly increase the effort while simultaneously reduce reproducibility. We opted for a strategy we believed would yield the most comprehensive results. Next, we consulted only two electronic databases, PubMed and the Cochrane Library, recognizing that other databases and libraries may include additional studies relevant to this work. However, PubMed was chosen because it is the most popular and likely most relevant database, with 23 million references in 2014 [22]. The Cochrane Library, with 800,000 references in 2014 [22], provides high-quality, peer-reviewed evidence in medical research and other services, mostly in the form of reviews. Google Scholar was not included because it returned almost identical results to PubMed. Furthermore, most of the studies are quite old. More than half of them were published before 1990, and most of them date back to the 1970s. Also, there were also almost no studies from the 1960s and earlier. There could be several reasons for this: English as a scientific language may not have been so common, the databases have not yet fully captured older or very old papers electronically, or the publications are not available in full text (only abstracts), so they did not meet our selection criteria. Another reason could be that the Hemox Analyzer, which greatly simplified the often complex measurements, was not introduced before the 1980s.

## Conclusions

The search yielded a manageable number of studies with statistically significant effects of dozens of drugs on the ODC. In most cases, these effects were a right-ward shift, which would result in improved tissue oxygenation. This information may be relevant to conditions such as pulmonary diffusion disorders, peripheral vascular diseases, or coronary artery diseases. However, the research is incomplete and highly fragmented, and awareness of the therapeutic implications is still marginal.

## Supplementary Information

Below is the link to the electronic supplementary material.

Supplementary file1 (DOCX 12 KB)

## Data Availability

No datasets were generated or analysed during the current study.

## Abbreviations

2,3-BPG: 2,3-Bisphosphoglycerate
Hb: Hemoglobin
ODC: Oxygen dissociation curve
SO_2_: Oxygen saturation
