# Supplementary material for: Effects of drugs on the oxygen dissociation curve—a scoping review
Source: Eur J Clin Pharmacol. 2024 Nov 25;81(2):227–36. doi: 10.1007/s00228-024-03781-8 (PMC11717808; doi:10.1007/s00228-024-03781-8)
Supplement: Supplementary file 1 — Supplementary file1 (DOCX 12 KB) [file 228_2024_3781_MOESM1_ESM.docx]

**Supplemental Information**

Institutional subscriptions to online journals: https://ezb.ur.de/ezeit/fl.phtml?bibid=UBI&colors=7&lang=en&bibid=UBI
